# Chromosome Synapsis and Recombination in Male Hybrids between Two Chromosome Races of the Common Shrew (*Sorex araneus* L., Soricidae, Eulipotyphla)

**DOI:** 10.3390/genes8100282

**Published:** 2017-10-20

**Authors:** Nadezhda M. Belonogova, Andrei V. Polyakov, Tatyana V. Karamysheva, Anna A. Torgasheva, Jeremy B. Searle, Pavel M. Borodin

**Affiliations:** 1 Institute of Cytology and Genetics, Russian Academy of Sciences, Siberian Department, Novosibirsk 630090, Russia; belon@bionet.nsc.ru (N.M.B.); polyakov@bionet.nsc.ru (A.V.P.); kary@bionet.nsc.ru (T.V.K.); torgasheva@bionet.nsc.ru (A.A.T.); 2 Novosibirsk State University, Department of Cytology and Genetics, Novosibirsk 630090, Russia; 3 Department of Ecology and Evolutionary Biology, Cornell University, Ithaca, NY 14853, USA; jeremy.searle@cornell.edu

**Keywords:** common shrew, meiotic chromosomes, *Sorex araneus*, synaptonemal complex, hybrids, MLH1

## Abstract

Hybrid zones between chromosome races of the common shrew (*Sorex araneus*) provide exceptional models to study the potential role of chromosome rearrangements in the initial steps of speciation. The Novosibirsk and Tomsk races differ by a series of Robertsonian fusions with monobrachial homology. They form a narrow hybrid zone and generate hybrids with both simple (chain of three chromosomes) and complex (chain of eight or nine) synaptic configurations. Using immunolocalisation of the meiotic proteins, we examined chromosome pairing and recombination in males from the hybrid zone. Homozygotes and simple heterozygotes for Robertsonian fusions showed a low frequency of synaptic aberrations (<10%). The carriers of complex synaptic configurations showed multiple pairing abnormalities, which might lead to reduced fertility. The recombination frequency in the proximal regions of most chromosomes of all karyotypes was much lower than in the other regions. The strong suppression of recombination in the pericentromeric regions and co-segregation of race specific chromosomes involved in the long chains would be expected to lead to linkage disequilibrium between genes located there. Genic differentiation, together with the high frequency of pairing aberrations in male carriers of the long chains, might contribute to maintenance of the narrow hybrid zone.

## 1. Introduction

The role of chromosome rearrangements in speciation is a subject of discussion. The traditional underdominance model suggests that intertaxon hybrids heterozygous for chromosome rearrangements should display various irregularities in meiotic chromosome pairing, recombination and segregation, and therefore become completely or partially sterile, due to meiotic arrest or formation of unbalanced gametes [1]. This model faces serious theoretical difficulties, because rearrangements causing sterility have a very low chance to spread in populations [2]. A more convincing modification of this model presumes that accumulation of several different selectively neutral chromosome rearrangements in different geographically isolated populations may lead to chromosomal incompatibility in the hybrids and their sterility [3,4,5]. Gene flow between chromosomally different populations, races or species may also be restricted, due to recombination suppression near the breakpoints of the chromosome rearrangements [6]. 

The common shrew, *Sorex araneus*, is a model species to examine various scenarios of chromosomal speciation. This species shows a high level of chromosome variation. The diploid chromosome number (2n) varies from 20 to 33, with the number of autosomal arms the same all over the species range (FNa = 40). It has been suggested that the ancestral karyotype of the common shrew consisted mostly of uniarmed (acrocentric) chromosomes [7]. The consecutive fixation of various centric or Robertsonian fusions (Rbs) and whole-arm reciprocal translocations (WARTs) gave rise to a wide variety of combinations of biarmed (metacentric) and acrocentric chromosomes in extant chromosome races. About 70 chromosome races have been described so far [8]. These races are distributed in a parapatric fashion, and generate many hybrid zones. The zones differ from each other in the complexity of meiotic configurations in the hybrids. In some zones, the hybrids carry only trivalents involving metacentric chromosomes paired with twin acrocentrics, while in the other zones, hybrids carrying chains of up to eleven chromosomes are observed [9]. 

**Figure 1:**
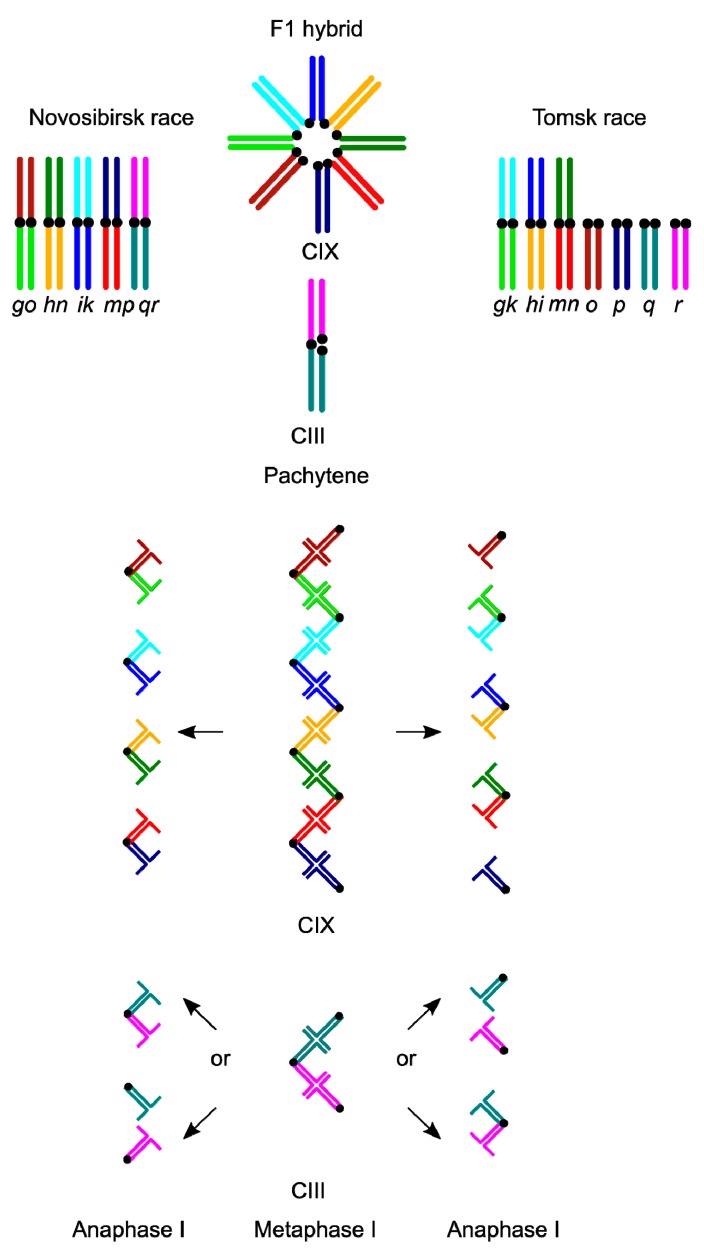
Expected meiotic configurations of the shrews of Novosibirsk and Tomsk races and their hybrids at pachytene (above) and later in the first meiotic division (below). Homologous arms are labelled and colour coded. Only balanced anaphase I complements are shown.

The hybrid zone between the Novosibirsk and Tomsk races is of special interest. The races differ for a series of chromosome rearrangements (Figure 1, Supplementary Figure S1). They form a narrow hybrid zone and generate hybrids with both simple and complex synaptic configurations [10,11]. The simple configurations are trivalents, i.e., chains of three (CIII), involving metacentric and twin acrocentric chromosomes. The complex chain configurations occur due to synapsis of eight (CVIII) or nine (CIX) metacentric and acrocentric chromosomes with monobrachial homology (Figure 1). The chromosomes involved in a CIX chain form clines of about 9 km width on average, while the cline for the CIII chromosomes is 53 km wide. Thus, the cline widths are inverse to the complexity of synaptic configurations, and the probability of meiotic errors [11].

Studies on gene flow across this zone have given contradictory results. On the one hand, analysis of population structure using several microsatellite markers revealed rather weak differentiation between the races; genetic differences between the Novosibirsk and Tomsk races were less than the inter-population differences within each race [12]. On the other hand, significant difference in morphology between the races has been demonstrated. The Novosibirsk shrews and the hybrids are significantly smaller than the Tomsk shrews [13]. Phenotypic differentiation in the geometric shape of skulls and mandibles across this zone was found to be greater than expected under the assumption of unrestricted gene flow during the estimated time since contact [14]. Interestingly, the morphological clines coincide with the CIX chromosome clines. The difference in the cline widths is parallel with the difference in expected level of meiotic abnormalities in CIX and CIII chromosome carriers [11,14].

In order to determine the role of chromosomal heterozygosity in the restriction of gene flow between the races, we examined chromosome pairing and recombination in shrews of various karyotypes trapped in the hybrid zone between the Tomsk and Novosibirsk chromosome races. To visualise synaptonemal complexes (SC) and the sites of crossing over, we used fluorescently-labelled antibodies to Synaptonemal Complex Protein 3 (SYCP3), a protein of the lateral elements of the SC, and MutL Homolog 1 (MLH1), a mismatch repair protein marking about 90% of mature recombination nodules. This approach has been successfully applied for mapping the sites of recombination in several species of mammals [15,16,17], including the common shrew [18].

## 2. Materials and Methods

### 2.1. Animals

Sixteen juvenile male shrews were used in this study. The animals were collected with Sherman live traps in the hybrid zone between the Novosibirsk and Tomsk chromosome races (54°47′ N; 83°25′ E) located 30 km southeast from Novosibirsk city (West Siberia, Russia) [10]. Experiments described in this manuscript were carried out in accordance with the approved national guidelines for the care and use of laboratory animals. All experiments were approved by the ethics committee on animal care and use of the Institute of Cytology and Genetics of the Siberian Department of the Russian Academy of Sciences, Russia (approval No. 35 of 26 October 2016).

### 2.2. Karyotyping

Bone marrow chromosome spreads were prepared according to Searle [19], and Giemsa band (G-band) stained [20]. We followed the chromosome nomenclature suggested by Searle et al. [21]. Chromosome arms and acrocentric chromosomes were presented by italicised letters. Metacentric chromosomes were described by two letters (the first for the long arm, the second for the short arm). Heterozygotes for a metacentric and twin acrocentric chromosomes, for example *qr* and *q*, *r*, are described as *q*/*r*. Heterozygotes forming larger meiotic chains are described by a list of single chromosomes involved in chain in a way to indicate the pairing with a ‘/’ between the chromosomes. All animals used in this study were homozygous for the chromosomes *af*, *bc*, *jl*, *tu*, and had the sex trivalent X/Y_1_Y_2_. Karyotypic categories of the shrews were defined by the complement of diagnostic metacentric chromosomes: *go*, *ik*, *hn*, *mp *for the Novosibirsk race and *gk*, *hi*, *mn* for the Tomsk race (Figure 1). The karyotypes of all individuals examined are listed in Table 1.

### 2.3. Meiotic Chromosome Preparation, Staining and Identification

Spermatocyte spreads were prepared from testis tissue using the drying-down technique [22]. A subset of cell spreads was processed for imaging by electron microscopy. Cells were stained with silver nitrate [23], and slides were covered with plastic film. After light microscopic examination, cells were transferred to specimen grids, examined, and photographed with an electron microscope JEM-100 (JEOL, Tokyo, Japan) at 80 kV. The remainder of cell spreads was processed for imaging by fluorescent microscopy. The immunostaining protocol was similar to that of Anderson et al. [16]. The slides were incubated for 2 h at 37 °C with a rabbit polyclonal antibody against rat lateral element protein SYCP3 (Abcam, Cambridge, UK) diluted to a concentration of 1:1000, and a mouse monoclonal antibody to mouse mismatch repair protein MLH1 (Abcam) at 1:50 dilution in 3% bovine serum albumin (BSA) in phosphate buffered saline (PBS). Slides were washed in 1× PBS and incubated for 40 min at 37 °C with donkey anti-rabbit Cy3-conjugated antibodies (Jackson ImmunoResearch Laboratories, Inc., West Grove, PA, USA) at 1:200 dilution and goat anti-mouse Fluorescein isothiocyanate (FITC)-conjugated antibodies (Jackson) at 1:400 dilution. Slides were washed with PBS, dried, and mounted in Vectashield with DAPI (Vector Laboratories, Burlingame, CA, USA) to stain DNA and reduce fluorescence fading.

The preparations were visualised with an Axioplan 2 imaging microscope (Carl Zeiss, Oberkochen, Germany) equipped with a CCD camera (CV M300, JAI Corporation, Yokohama, Japan), CHROMA filter sets and ISIS4 image-processing package (MetaSystems, Altlußheim, Germany). Brightness and contrast of all images were enhanced using Corel PaintShop Photo Pro X3 (Corel Corporation, Ottawa, ON, Canada). Only cells containing complete sets of chromosomes were analysed. MLH1 signals were only scored if they were localised on an SC. Each chromosome arm was identified by its specific DAPI pattern, according to Belonogova et al. [24] and relative size. The centromere position for each SC was identified by DAPI-banding. In some meiotic configurations, we observed pericentromeric regions unlabelled with SYCP3. In these configurations, the most proximal end of the SYCP3-labelled SC was recognised as the centromere (in accordance with DAPI banding). The length of each SYCP3-labelled chromosome arm was measured in micrometres using MicroMeasure 3.3 [25] and the positions of MLH1 foci were recorded in relation to the centromere. Total MLH1 count was scored in the cells without synaptic abnormalities, i.e., having completely paired bivalents/multivalents and clear MLH1 staining. To generate recombination maps, we used all completely paired bivalents/multivalents without synaptic abnormalities that contained at least one MLH1 focus (regardless of the synaptic state of other arms in the cell). MLH1 focus distances from the centromere were not measured for chromosomes where the centromeres were not clearly visible (for example, due to overlapping of chromosomes). We calculated the absolute position of each MLH1 focus multiplying the relative position of the focus by the absolute length for the appropriate chromosome arm averaged for all karyotypes. Statistica 6.0 software package (StatSoft, Tulsa, OK, USA) was used for descriptive statistics.

## 3. Results

### 3.1. Karyotype Variability in the Hybrid Zone

Table 1 shows karyotypes and synaptic characteristics of the male shrews subjected to SC analysis. Among them, we detected ten carriers of diagnostic chromosomes of the Novosibirsk race, three carriers of diagnostic chromosomes of Tomsk race and three complex heterozygotes for these chromosomes. 

The karyotype variability in the hybrid zone makes impossible reliable discrimination between “purebred” representatives of parental races, F_1_ hybrids between them, and recombinant progeny of the F_1_ hybrids and their backcrosses to parental races. For this reason, we grouped the specimens according to complexity of synaptic configurations occurring in meiosis, rather than to their origin inferred from the sets of diagnostic chromosomes. Among the carriers of the Novosibirsk karyotype, five specimens were simple heterozygotes for *q/r*, four for *m/p*, two for *g/o*. One of the carriers of the Tomsk race diagnostic chromosomes was heterozygous for *q/r*, one was homozygous for metacentric chromosome *qr*, typical for the Novosibirsk race, and one was homozygous for acrocentric chromosomes *q* and *r*, typical for the Tomsk race. One of the complex heterozygotes was also homozygous for the Novosibirsk variant of chromosome *qr*, while another two were heterozygous for *q/r. *Among the complex heterozygotes, one had CIX and two had CVIII. The chromosome chain in CVIII specimens was truncated because they were homozygous for acrocentric chromosome *p*.

### 3.2. Synaptic Aberrations

**Figure 2:**
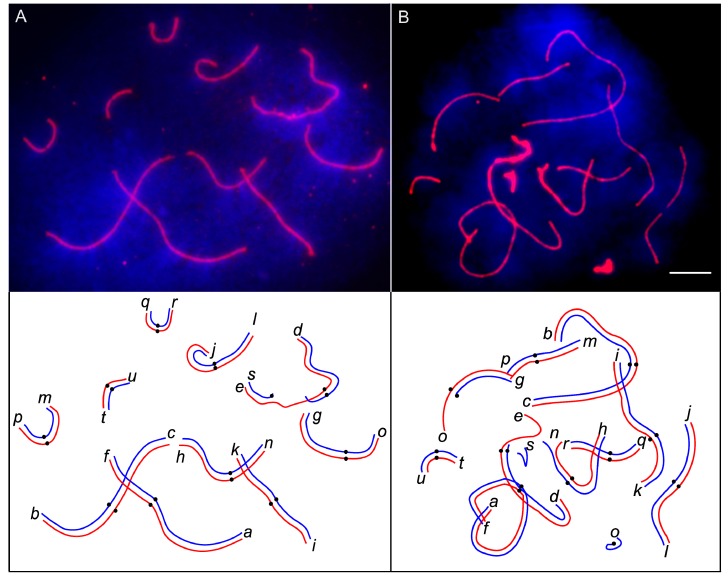
Fluorescent micrographs and interpretative diagrams of pachytene spermatocytes of a normal homozygote (**A**) and a CIII (*g/o*) heterozygote (**B**) after immunolocalisation of SYCP3 (red) and DAPI counterstaining (blue). Centromeres are marked with black circles on the diagrams. Letters indicate distal ends of the chromosome arms. Scale bar: 5 µm.

In the majority of pachytene cells of the homozygotes and the simple heterozygotes for one or two CIII, we observed orderly paired autosomes. The sex trivalent X/Y_1_Y_2_ followed its usual pairing pattern [18,26,27]: the autosomal part of Xq (arm *d*) and the whole Y_2_ were completely synapsed, while the Y_1_ (arm *s*) was partly paired with the end of Xp (arm *e*) in a short pseudoautosomal region (Figure 2A). Pachytene cells of simple heterozygotes for one or two CIII usually contained completely synapsed trivalents with or without formation of side arms between pericentromeric regions of the twin acrocentrics. Pairing abnormalities, such as partial asynapsis of the chromosome ends in the bivalents and pericentromeric regions of the twin acrocentrics in the trivalents, or complete asynapsis of one of the twin acrocentrics (Figure 2B), were rare in most specimens of these karyotypes. Two specimens, #5 and #13, demonstrated a rather high frequency of pairing abnormalities, but only a few cells were suitable for SC analysis in these specimens (Table 1). The differences in frequency of pairing abnormalities between the homozygotes and simple heterozygotes for one or two CIII were not significant (Fisher’s exact test *p* > 0.05). 

**Figure 3:**
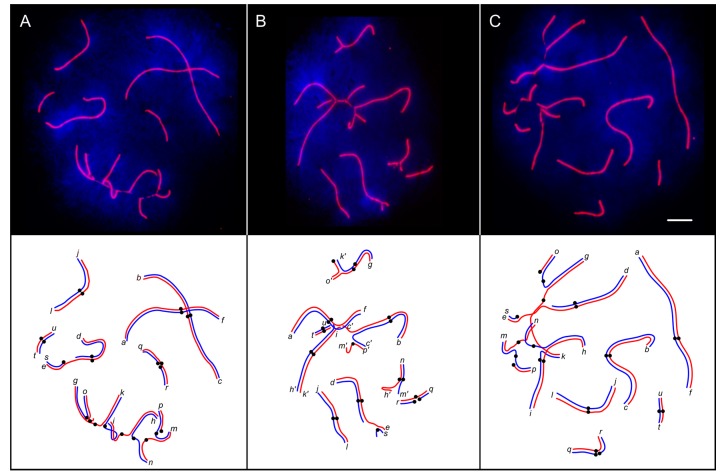
Fluorescent micrographs and interpretative diagrams of pachytene spermatocytes of a CVIII heterozygote after immunolocalisation of SYCP3 (red) and DAPI counterstaining (blue). Centromeres are marked with black circles on the diagrams. Letters indicate distal ends of the chromosome arms. A prime following the letter indicates that it is paired non-homologously or completely unpaired. Scale bar: 5 µm. (**A**) homologous synapsis of all arms in a CVIII multivalent with partial asynapsis around the centromeres; (**B**) dissociation of a CVIII multivalent into novel shorter configurations *gk/go/o*, *bc/bc/mp*, *hn/mn*, *hi/ki*, and univalent *p*; (**C**) association between the sex trivalent and the CVIII configuration.

About half of the pachytene cells of the males with a CVIII configuration and most pachytene cells of the male with a CIX configuration showed synaptic abnormalities (Table 1, Figure 3). The difference in the frequency of synaptic aberrations between CVIII and CIX karyotypes, as well as the differences between complex heterozygotes, on the one hand, and homozygotes and simple heterozygotes, on the other hand, were significant (Fisher’s exact test *p* < 0.01). 

**Figure 4:**
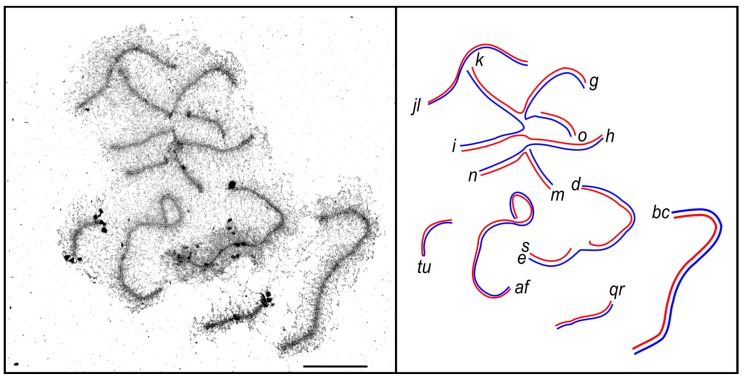
Electron micrograph and interpretative diagram of a pachytene spermatocyte of a CVIII heterozygote after AgNOR staining. Letters indicate chromosomes and chromosome arms. Scale bar: 5 µm.

A considerable part of the pericentromeric SCs of the multivalents usually appeared unpaired, and had a reduced intensity of SYCP3 and AgNOR staining. We often observed large unstained gaps in these regions, even when all arms of the multivalents were synapsed (Figure 3A and Figure 4). Matveevsky et al. [28] also observed such gaps in tetravalents of hybrid male shrews. We suggest that the gaps are a spreading artefact. Resistance of single axial elements to the spreading in the multivalents is weaker than that of double-paired elements of the bivalents. For this reason, the centromeric gaps are more pronounced in multivalents.

Complete asynapsis of one or more chromosome arms resulted in dissociation of the expected CVIII and CIX configurations into two or more novel configurations. For example, we observed g*k/go/o*, *hn/mn*, and *hi/ki* with the shared arms paired homologously, and other arms were involved in non-homologous synapsis (Figure 3B).

CVIII and CIX multivalents were often associated with the configurations formed by invariable chromosomes. Most often, we observed an association between the CIX and the sex trivalent (Figure 3C). The remaining nuclei contained multivalents or their elements associated or paired with *af *or *bc* bivalents, or with both of them (Figure 3B). Chromosomes *jl*, *qr*, and *tu* were seldom found attached to the chain.

**Figure 5:**
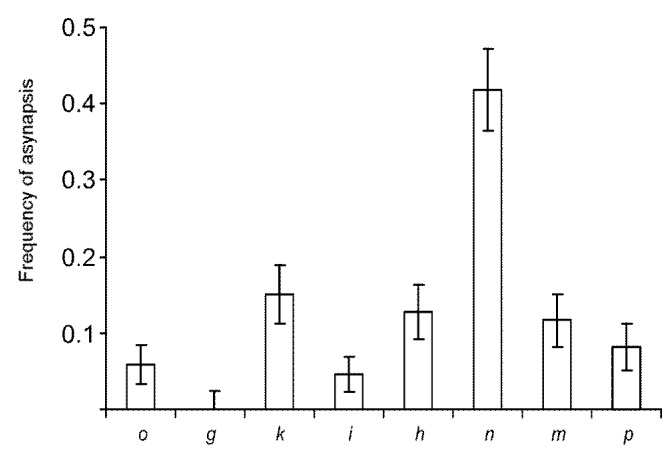
Frequency of asynapsis in the chromosome arms involved in the CIX configuration. Error bars show standard deviation. Letters indicate chromosome arms.

The arms that contribute to the CIX configuration varied in the frequency of asynapsis (Figure 5). We did not observe a pairing failure in the arm *g*, while the arm *n* was unpaired significantly more often than any other arm (Fisher’s exact test with Bonferroni correction on multiple testing *p* < 0.002). There was no correlation between the frequency of asynapsis and the arm size (*p* > 0.05). 

### 3.3. MLH1 Foci Number and Distribution

**Figure 6:**
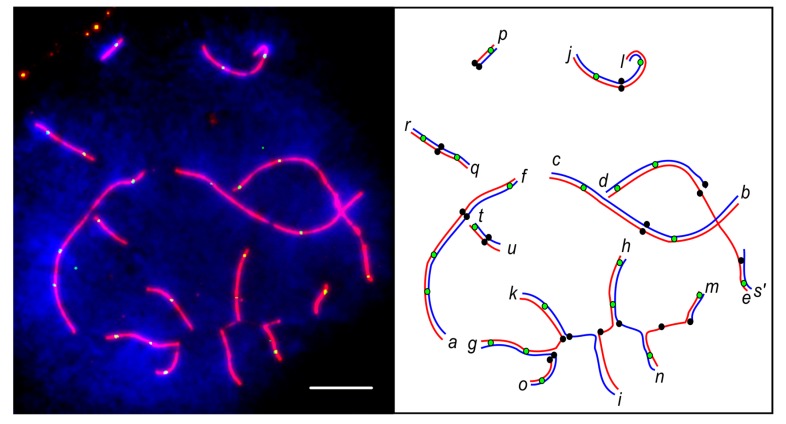
Fluorescent microphotograph and interpretative diagram of pachytene spermatocyte of a CVIII heterozygote after immunolocalisation of SYCP3 (red) and MLH1 (green), and DAPI counterstaining (blue). Centromeres are marked with black circles on the diagram. MLH1 foci marked with green circles. Letters indicate distal ends of the chromosome arms. Scale bar 5 µm.

We used MLH1 foci as markers of recombination points along the SCs (Figure 6). Table 2 shows the average number of MLH1 foci per cell for all shrews examined. We also included in Table 2 our estimates of the expected number of obligate chiasmata for each karyotype. We expected that at least one chiasma per chromosome is necessary for a proper segregation of the chromosomes involved into bivalents and one per arm for an orderly segregation of trivalents and longer multivalents. The male with a CIX configuration, which showed the highest frequency of synaptic abnormalities, had the lowest MLH1 foci number among the shrews examined. It was lower than the expected number of obligate chiasmata (*p *= 0.04). In the other shrews, the observed number of MLH1 foci was higher than the expected number of obligate chiasmata (all *p* values < 10^−9^).

Supplementary Table S1 shows the mean number of MLH1 foci per chromosome arm in different karyotypes. Despite a high frequency of synaptic aberration detected in CVIII carriers, we did not find a significant reduction of MLH1 foci number either for the arms of invariable chromosomes, or for the arms of the chain forming chromosomes (*p* > 0.05 in each pairwise comparison). 

**Figure 7:**
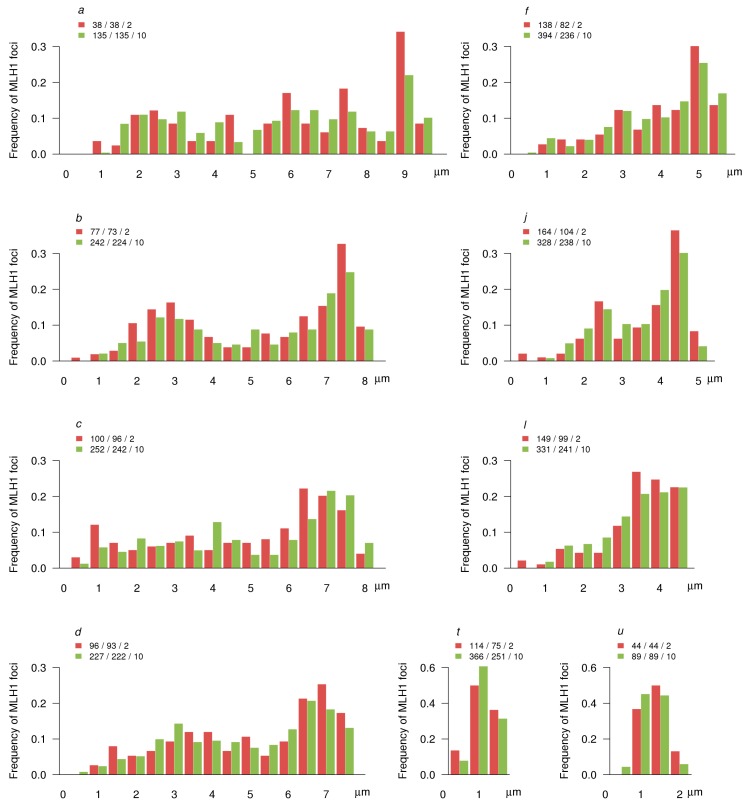
Distribution of MLH1 foci along the arms of invariable chromosomes. The X-axis shows the position of MLH1 foci in relation to the centromere (marked by 0) for the synaptonemal complexes (SC) of the particular arm (indicated at the top of each graph) in bivalent/trivalent carriers (red) and complex heterozygotes with a CVIII configuration (green). The labels on the X-axis are separated by 1 µm of the SC. The Y-axis indicates the frequency of MLH1 foci in each 0.5 µm interval. The numbers in the legends above each plot show the number of MLH1 foci/arms/specimens plotted.

**Figure 8:**
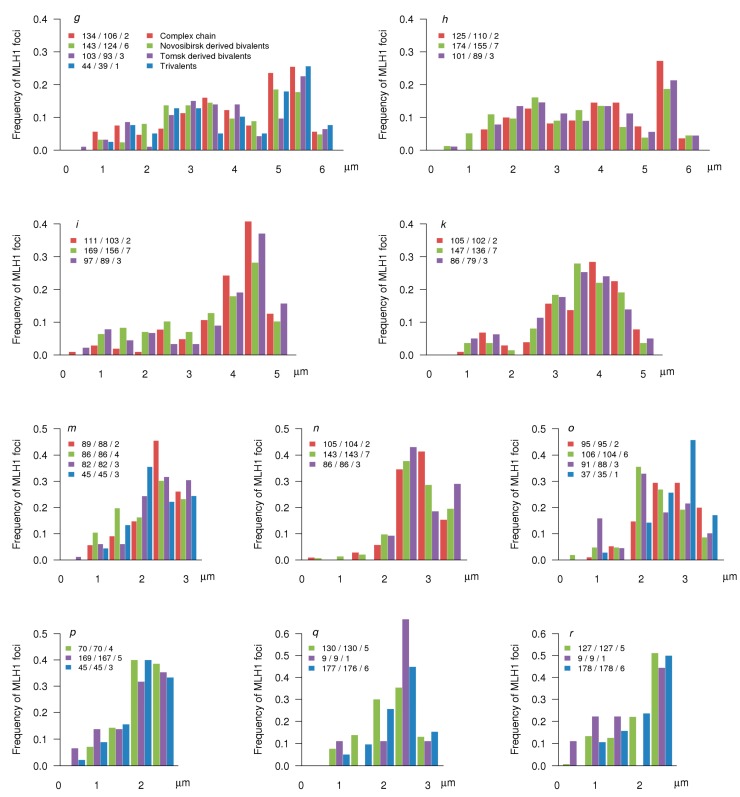
Distribution of MLH1 foci along the arms of variable chromosomes. The X-axis shows the position of MLH1 foci in relation to the centromere (marked by 0) at the SC of particular arm (indicated at the top of each graph). The labels on the X-axis are separated by 1 µm of the SC. The Y-axis indicates the frequency of MLH1 foci in each 0.5 µm interval. The numbers in the legends above each plot show the number of MLH1 foci/arms/specimens plotted.

The distribution of MLH1 foci along the SCs of the Siberian shrews matched the pattern described for the shrews sampled from Oxford–Wirral hybrid zone [18]. For all chromosome arms examined, we observed pronounced peaks at the distal regions and a deficiency of the foci in pericentromeric regions (Figure 7 and Figure 8). Large and some medium sized chromosome arms showed a multi- or bi-modal MLH1 foci distribution. However, even in these arms, proximal peaks were lower than the distal ones. Medium and small sized arms, including those involved in the CVIII and the CIII, showed a gradual decrease of MLH1 foci number from distal to medial chromosome regions. The distribution of MLH1 foci along the invariable chromosomes was rather similar in the CVIII individual and the shrews homozygous for the chain forming chromosomes (Figure 7).

All chain forming arms except arms *g* and *h* contained very few MLH1 foci in their proximal regions. We observed an especially wide and deep pericentromeric valley of MLH1 frequency for arm *n* (Figure 8), characterised by the highest frequency of asynapsis in the CVIII (Figure 5).

Each arm displayed its individual pattern of MLH1 foci distribution. It was not substantially altered by the arm combination in the metacentric homozygotes. For example, the arm *g *showed a similar pattern in the *go* and *gk *combinations (Figure 8).

Having found extensive asynapsis in the pericentromeric regions in the chain configuration (Figure 3 and Figure 6), we expected to observe a substantial distalisation of the MLH1 focus distribution in the complex heterozygotes; this was not the case. Although in all arms compared, the distance between centromeres and MLH1 foci was slightly higher in complex heterozygotes (CVIII) than in metacentric homozygotes, the differences were not significant (*p* > 0.05) (Supplementary Figure S2). Apparently, recombination in the proximal regions is so strongly suppressed in normal homozygous karyotype that partial asynapsis in these regions does not make it any stronger.

## 4. Discussion

Here, we demonstrated a very high rate of synaptic aberration in male complex heterozygotes for chromosome rearrangements with monobrachial homology derived from the hybrid zone between the Novosibirsk and Tomsk chromosome races of the common shrew (Table 1) and strong recombination suppression in pericentromeric regions of most chromosomes. How may these factors affect gene flow across the hybrid zone? 

Partial or complete asynapsis of the chromosomes involved in the chain was the abnormality most often seen. Asynapsis is a well-known cause of impaired fertility due to meiotic arrest and germ cell loss [29]. Completion of synapsis at pachytene is monitored by a checkpoint mechanism, which triggers apoptosis in the cells with incomplete synapsis [30,31,32]. However, at least half of the germ cells in CVIII carriers showed normal chromosome synapsis and recombination. These cells can complete meiosis and form viable gametes. Earlier, Pavlova et al. [33,34] and Matveevsky et al. [28] detected normal pairing of the expected configurations at diakinesis–metaphase I and active sperm of normal morphology in male carriers of ring of four (RIV) and CXI from the Moscow–Neroosa and Moscow–Seliger hybrid zones, respectively. Studies in other hybrid zones of the common shrew suggest decreases in fertility in heterozygotes, particularly complex heterozygotes, but not to a massive degree [35,36,37,38]. Unfortunately, we were unable to obtain direct estimates of fertility of the shrews from the Novosibirsk–Tomsk hybrid zone. Breeding shrews in captivity is extremely difficult. Trapping adult male shrews during the breeding season (April–May) in this area is impossible, due to climatic conditions. However, the indirect evidence listed above indicates that despite a high frequency of pachytene cells with aberrant chromosome pairing, the male carriers of complex meiotic configuration generally can produce viable gametes, although likely in reduced numbers. 

Analysis of the distribution of molecular markers in the hybrid zones of the common shrew showed no evidence of reduced gene flow resulting from male hybrid sterility or reduced fertility. There was no difference in the degree of genetic differentiation within and between the Novosibirsk and Tomsk races; the markers located in common and race-specific chromosomes showed the same level of differentiation between the races [12]. No linkage disequilibrium was detected between the race specific Y chromosome haplotypes and the race specific autosome complement in the Novosibirsk–Tomsk hybrid zone [39]. These indirect data indicate that pairing abnormalities in hybrid CVIII and CIX carriers, detected in this study, do not lead to complete sterility of males. 

Female meiosis in the hybrids has not been studied yet, due to technical difficulties. However, studies on the other mammalian species indicate that female meiosis is less vulnerable to chromosomal heterozygosity and genetic incompatibility than male meiosis. It is also known that meiotic checkpoints in female germ cells are less sensitive to incomplete synapsis than in male germ cells (see [40] for review). For this reason, we suppose that the fertility in female hybrid shrews should be affected less than in males. 

The overall recombination rate was rather similar in all shrews examined, except the male CIX carrier showing a very high frequency of pairing aberration (Table 2). In the homozygous CIII and CVIII karyotypes, it was higher than the expected number of obligatory chiasmata sufficient for correct chromosome segregation at the first meiotic division (Table 2). Thus, correct segregation of chromosomes is possible in the male hybrids, though direct studies are needed to estimate the frequency of meiotic non-disjunction in this hybrid zone. Certainly, the narrowness of the Novosibirsk–Tomsk hybrid zone indicates that there is a degree of infertility associated with presence of the CVIII or CIX configuration in hybrids [11], either attributable to germ cell death (reduced reproductive lifespan of females, greater frequency of males with insufficient numbers of sperm) or non-disjunction (greater embryo death) [35].

Figure 1 shows the balanced segregation of the CIX, when all chromosomes derived from Tomsk race move to one pole and all Novosibirsk derived chromosomes to another pole. Of course, recombination in the terminal and medial regions of the chromosomes lead to the production of recombined chromosomes. However, a strong suppression of recombination around the centromeres, spanning from a quarter to a half of the arm length in the chain forming metacentric chromosomes, enforces linkage disequilibrium between all parental alleles located in the pericentromeric regions. If in geographic isolation, chromosome races become fixed around the centromeres with alternative epistatically-interacting coadaptive genes, i.e., supergenes, then such supergenes could still be maintained after the races come into contact. Such pericentromeric supergenes have been found in several species of plants (see [41] and references therein). In the shrew hybrids, these supergenes could be dispersed over multiple pericentromeric regions of the chromosomes involved in the long meiotic chain, and therefore, would segregate as a united race specific “super-supergene”. It has been shown that the gene flow between mouse chromosome races is more strongly restricted near the centromeres of Robertsonian chromosomes than in other regions [42,43,44]. In the case of Novosibirsk–Tomsk hybrid zone, we would also expect strongly restricted gene flow in the proximal regions. This reduction in gene flow is probably very difficult to detect with the set of chromosome-specific microsatellite markers available at present in the common shrew [12]. More markers with known subchromosomal localisation have to be developed.

Linkage disequilibrium, due to crossover suppression in pericentromeric regions in the complex chromosome heterozygotes occurring in the hybrid zone, might keep together race-specific alleles controlling morphological traits, and thus, contribute to the phenotypic differentiation of the parental races [13,14]. Linkage disequilibrium for genes controlling local adaptations may also affect the width of clines for the chromosomes containing such genes. Polyakov at al. [45] demonstrated altitudinal partitioning of the Novosibirsk and Tomsk races. This partitioning marks a border between two rather different biotopes: forest steppe at low altitude, and taiga at the high. It has been shown that the structure of the cline for the CIX chromosomes closely follows that predicted by a model including altitude, while the CIII cline does not fit it so well [11]. 

In conclusion, the meiotic studies of hybrids from the Novosibirsk–Tomsk hybrid zone of the common shrew show indications that both fertility reduction and recombination suppression may contribute to reduced gene flow, in line with previous findings for the best studied house mouse chromosome hybrid zone [43]. Together, the common shrew and house mouse have provided unusually detailed vignettes of the impacts of heterozygosity of Robertsonian chromosomes in nature, of relevance to our understanding of chromosomal rearrangements in differentiation and speciation [4]. Given that hybrid karyotypes have very different properties in other known hybrid zones of the common shrew [12], this species is an exceptional system to extend, even further, our understanding of the meiotic properties of a wide range of Robertsonian heterozygotes.

## Figures and Tables

**Table 1:** The frequency of synaptic aberrations.

ID	Synaptic Type (Autosomes)	Diagnostic Chromosome Set	Karyotype	2n	Number of Cells Scored	Frequency of Cells with Synaptic Aberrations
1	Bivalents only	Novosibirsk	*go*, *hn*, *ik*, *mp*, *qr*	21	29	0.07
2	Bivalents only	Novosibirsk	*go*, *hn*, *ik*, *mp*, *qr*	21	67	0.03
3	Bivalents only	Novosibirsk	*go*, *hn*, *ik*, *mp*, *qr*	21	31	0.06
4	Bivalents only	Tomsk	*gk*, *hi*, *mn*, *o*, *p*, *qr*	23	76	0.00
5	Bivalents only	Tomsk	*gk*, *hi*, *mn*, *o*, *p*, *q*, *r*	25	13	0.23
6	Single trivalents CIII	Tomsk	*gk*, *hi*, *mn*, *o*, *p*, *q/r*	24	74	0.07
7	Single trivalents CIII	Novosibirsk	*go*, *hn*, *ik*, *mp*, *q/r*	22	44	0.00
8	Single trivalents CIII	Novosibirsk	*g/o*, *hn*, *ik*, *mp*, *qr*	22	21	0.10
9	Single trivalents CIII	Novosibirsk	*go*, *hn*, *ik*, *m/p*, *qr*	22	78	0.03
10	Double trivalents CIII	Novosibirsk	*go*, *hn*, *ik*, *m/p*, *q/r*	23	21	0.10
11	Double trivalents CIII	Novosibirsk	*go*, *hn*, *ik*, *m/p*, *q/r*	23	19	0.00
12	Double trivalents CIII	Novosibirsk	*g/o*, *hn*, *ik*, *mp*, *q/r*	23	94	0.10
13	Double trivalents CIII	Novosibirsk	*go*, *hn*, *ik*, *m/p*, *q/r*	23	12	0.33
14	Complex chains CVIII	Hybrid	*o/og/gk/ki/ih/hn/nm/m*, *p*, *qr*	23	69	0.48
15	Complex chains CVIII + CIII	Hybrid	*o/og/gk/ki/ih/hn/nm/m*, *p*, *q/r*	24	39	0.44
16	Complex chains CIX + CIII	Hybrid	*o/og/gk/ki/ih/hn/nm/mp/p*, *q/r*	23	126	0.79

**Table 2:** Average number of MLH1 foci per cell (scored in the cells without synaptic abnormalities) and expected number of obligate chiasmata in the shrews of different karyotypes.

ID	Synaptic Type (Autosomes)	Karyotype	Number of Cells Scored	Average Number of MLH1 Foci per Cell ± S.D.	Expected Number of Obligate Chiasmata
1	Bivalents only	*go*, *hn*, *ik*, *mp*, *qr*	6	18.7 ± 3.1	11
2	Bivalents only	*go*, *hn*, *ik*, *mp*, *qr*	18	18.6 ± 3.3	11
4	Bivalents only	*gk*, *hi*, *mn*, *o*, *p*, *qr*	6	19.5 ± 2.4	12
5	Bivalents only	*gk*, *hi*, *mn*, *o*, *p*, *q*, *r*	7	19.8 ± 2.1	13
6	Single trivalents CIII	*gk*, *hi*, *mn*, *o*, *p*, *q/r*	15	22.1 ± 3.7	13
10	Double trivalents CIII	*go*, *hn*, *ik*, *m/p*, *q/r*	14	20.6 ± 3.4	13
12	Double trivalents CIII	*go*, *hn*, *ik*, *m/p*, *q/r*	27	19.1 ± 1.9	13
14	Complex chains CVIII	*o/og/gk/ki/ih/hn/nm/m*, *p*, *qr*	19	21.1 ± 2.5	15
15	Complex chains CVIII + CIII	*o/og/gk/ki/ih/hn/nm/m*, *p*, *q/r*	17	22.1 ± 2.8	16
16	Complex chains CIX + CIII	*o/og/gk/ki/ih/hn/nm/mp/p*, *q/r*	5	14.1 ± 2.5	16

S.D.: standard deviation.

## Supplementary Materials

The following is available online at www.mdpi.com/2073-4425/8/10/282/s1. Table S1: Number of MLH1 foci per chromosome arm. Figure S1: G-banded karyotypes of the male shrews of Novosibirsk and Tomsk races and their hybrid; Figure S2: SC length and distance of MLH1 foci from centromeres of the variable chromosome arms involved in different synaptic configurations.
