# Supplementary material for: Chromosome Synapsis and Recombination in Male Hybrids between Two Chromosome Races of the Common Shrew (Sorex araneus L., Soricidae, Eulipotyphla)
Source: Genes (Basel). 2017 Oct 20;8(10):282. doi: 10.3390/genes8100282 (PMC5664132; doi:10.3390/genes8100282)
Supplement: Supplementary file 1 [file genes-08-00282-s001.zip › Supplementary Figure S1.pdf]

Supplementary Figure S1.
G-banded karyotypes of the male shrews of Novosibirsk and Tomsk races and their hybrid.

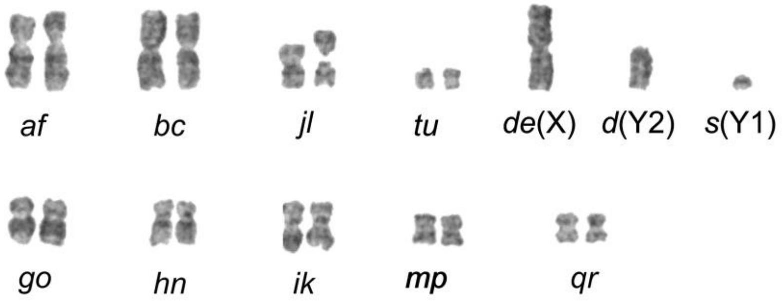

Novosibirsk race

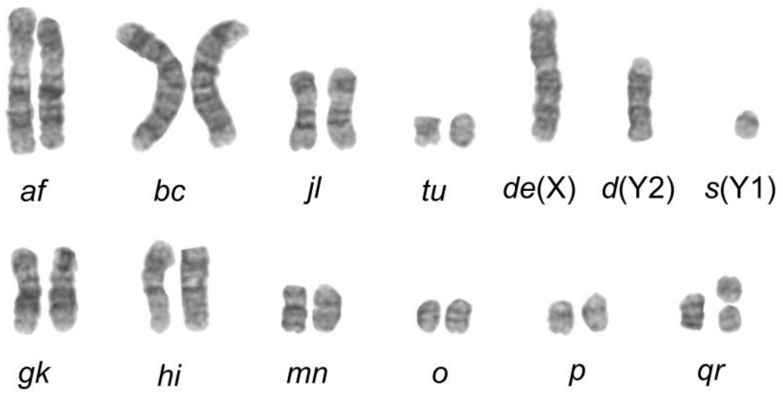

Tomsk race

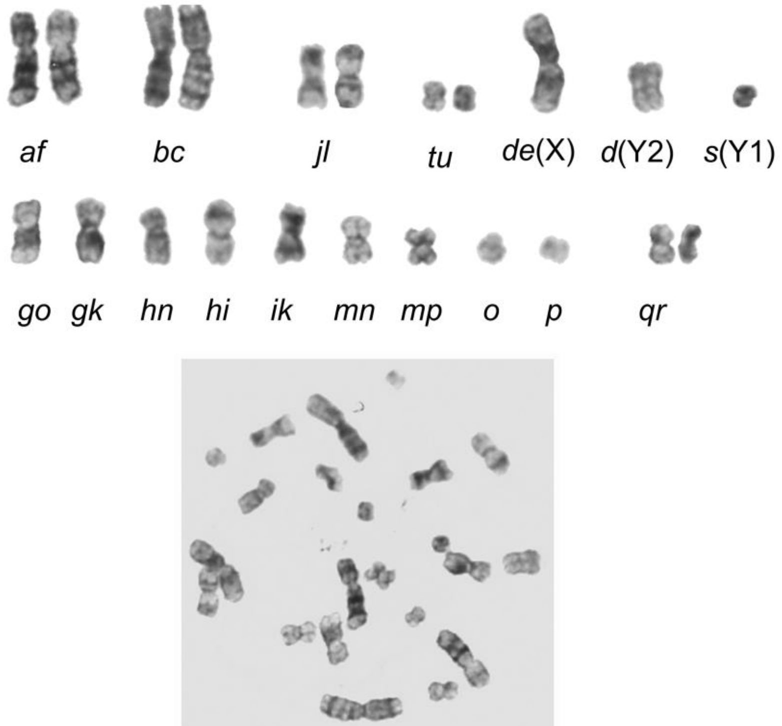

Hybrid between Novosibirsk and Tomsk races
