# Supplementary material for: Chromosome Synapsis and Recombination in Male Hybrids between Two Chromosome Races of the Common Shrew (Sorex araneus L., Soricidae, Eulipotyphla)
Source: Genes (Basel). 2017 Oct 20;8(10):282. doi: 10.3390/genes8100282 (PMC5664132; doi:10.3390/genes8100282)

Supplementary Figure S2.

SC length and distance of MLH1 foci from centromeres of the variable chromosome arms involved in different synaptic configurations.

Whole columns show average SC lengths of the chromosome arms from centromeres (bottom) to telomeres (top). Horizontal lines show mean distances of MLH1 foci from centromeres. The caption show colour codes of synaptic configurations. Numbers below each column indicate the number of arms measured (top line) and the arms containing MLH1 foci . Bars show S.E.

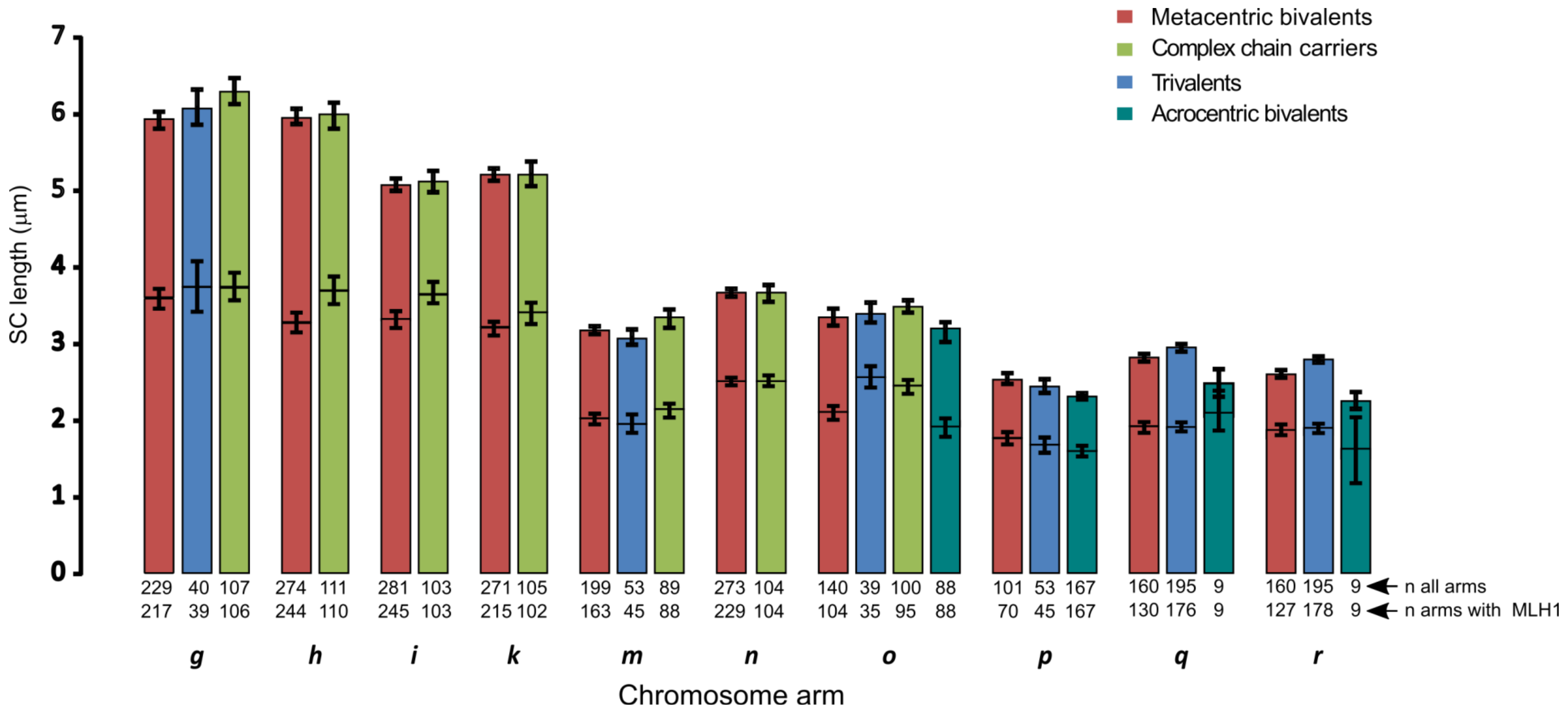

Supplement: Supplementary file 1 [file genes-08-00282-s001.zip › Supplementary Figure S2.pdf]
