# Supplementary material for: Chromosome Synapsis and Recombination in Male Hybrids between Two Chromosome Races of the Common Shrew (Sorex araneus L., Soricidae, Eulipotyphla)
Source: Genes (Basel). 2017 Oct 20;8(10):282. doi: 10.3390/genes8100282 (PMC5664132; doi:10.3390/genes8100282)
Supplement: Supplementary file 1 [file genes-08-00282-s001.zip › Supplementary Table S1.pdf]

Supplementary Table S1. Number of MLH1 foci per chromosome arm

Arm	Synaptic type	Number of MLH1 foci		Number of chromosomes examined	Number of animals
		Mean	S.D.		
Invariable chromosomes					
a	Met hom[a]	1.67	0.63	84	7
	CVIII[b]	1.90	0.61	30	2
b	Met hom	1.40	0.54	84	7
	CVIII	1.52	0.57	29	2
c	Met hom	1.46	0.63	85	7
	CVIII	1.45	0.57	29	2
d	Met hom	1.53	0.61	89	7
	CVIII	1.50	0.56	36	2
f	Met hom	1.01	0.40	84	7
	CVIII	1.00	0.46	30	2
j	Met hom	1.01	0.41	81	7
	CVIII	0.89	0.32	36	2
l	Met hom	0.94	0.33	81	7
	CVIII	0.92	0.37	36	2
t	Met hom	0.32	0.47	89	7
	CVIII	0.34	0.48	35	2
u	Met hom	0.59	0.5	89	7
	CVIII	0.66	0.48	35	2
Chromosomes involved in CVIII					
g	Met hom	1.18	0.54	57	6
	CIII[c] (g/o)	1.12	0.43	26	1
	CVIII	1.29	0.58	34	2
h	Met hom	0.99	0.46	85	7
	CVIII	1.13	0.42	32	2
i	Met hom	1.00	0.42	81	7
	CVIII	0.94	0.34	35	2
k	Met hom	0.88	0.40	83	7
	CVIII	1.03	0.39	34	2
m	Met hom	0.77	0.43	73	6
	CIII (m/p)	0.79	0.43	14	1
	CVIII	0.92	0.37	36	2
n	Met hom	0.90	0.31	89	7
	CVIII	0.94	0.23	36	2
o	Met hom	0.73	0.45	37	3
	CIII (g/o)	0.88	0.33	26	1
	Acro hom[d]	0.93	0.38	27	3
	CVIII	0.97	0.17	33	2
p	Met hom	0.65	0.48	51	3
	CIII (m/p)	0.79	0.43	14	1
	Acro hom	0.97	0.26	60	5
Chromosomes involved in CIII					
q	Met hom	0.75	0.48	48	4
	CIII	0.83	0.38	72	4
	Acro hom	1.00	-	6	1
r	Met hom	0.83	0.42	48	4
	CIII	0.86	0.35	72	4
	Acro hom	1.00	-	6	1

a – metacentric homozygotes

b – CVIII carriers
c – CIII carriers
d – acrocentric homozygotes
